# Host Genetics and HIV-1: The Final Phase?

**DOI:** 10.1371/journal.ppat.1001033

**Published:** 2010-10-14

**Authors:** Jacques Fellay, Kevin V. Shianna, Amalio Telenti, David B. Goldstein

**Affiliations:** 1 Center for Human Genome Variation, Duke University School of Medicine, Durham, North Carolina, United States of America; 2 Institute of Microbiology, University of Lausanne, Lausanne, Switzerland; University of California San Diego, United States of America

## Abstract

This is a crucial transition time for human genetics in general, and for HIV host genetics in particular. After years of equivocal results from candidate gene analyses, several genome-wide association studies have been published that looked at plasma viral load or disease progression. Results from other studies that used various large-scale approaches (siRNA screens, transcriptome or proteome analysis, comparative genomics) have also shed new light on retroviral pathogenesis. However, most of the inter-individual variability in response to HIV-1 infection remains to be explained: genome resequencing and systems biology approaches are now required to progress toward a better understanding of the complex interactions between HIV-1 and its human host.

## Introduction

Many fundamental questions about how and why humans differ in their susceptibility to HIV-1 remain largely unanswered. For example, it has long been known that a fraction of the human population cannot be infected by HIV-1 [1], [2]. We still do not know, however, whether most of those who are resistant to infection are resistant due to innate or adaptive immunity, or to some other mechanism. Nor are the precise pathways that allow apparently permanent control of the virus amongst a subset of those that do become infected well understood. These questions are obviously central in the effort to develop effective strategies to combat HIV-1, and at their heart, they are genetic.

Until recently, our capacity to systematically address these issues was limited. But genomic analyses have advanced to the point that comprehensive or nearly comprehensive analyses of the role of genetic variation in viral control is now within reach. A series of genome-wide association studies has already provided a detailed description of how common variation influences control of HIV-1 [3]–[9]. More importantly, next-generation sequencing is now sufficiently advanced such that a dedicated effort to uncover the role of rare variation has become feasible. Coupling these new developments with rich cohorts being built under the auspices of international groupings such as the Center for HIV/AIDS Vaccine Immunology (CHAVI) and the International HIV Controllers Study, all ingredients are now available for drawing conclusive answers to these fundamental questions.

Here, we first review what is known about how genetic variation influences HIV-1 acquisition and control. We then describe new developments and argue that a concerted effort is now appropriate to bring these elements together to answer these outstanding questions decisively and draw the appropriate lessons for vaccine development as well as understanding of pathogenesis.

## Cohorts

### Cohorts for the Study of HIV-1 Acquisition

In various high-risk populations, there are reports of individuals that have been repeatedly exposed to HIV-1 and yet have not been infected. Exposure itself has been assessed in various ways, but in all cases it appears that portions of the population are protected. Perhaps the most striking example is the case of hemophilia: a vast majority of severe hemophilia A patients born before 1979 became HIV-1 seropositive, due to virtually universal exposure to contaminated batches of factor VIII concentrates. Still, about 5% of them remained seronegative [1]. Other examples of exceptional resistance to infection have been described in cohorts of men who have sex with men reporting high-risk behavior [10] and of female sex workers in Nairobi, Kenya [11]. Finally, virtually all infectious disease clinicians report individual patients with very high levels of exposure that did not become seropositive; a celebrity such as Sir Elton John also described himself as a lucky person that mysteriously avoided infection. The genetic analysis of well-characterized highly exposed, yet uninfected, individuals is thus essential. An alternative approach to study HIV-1 acquisition is to test for differences in allelic distribution between patients with HIV-1 and large cohorts of presumably uninfected controls, since the infected population will then be depleted in protective factors or enriched in alleles conferring enhanced susceptibility to infection. Many groups active in HIV-1 host genetics research have recently created the International HIV Acquisition Consortium to initiate such a study.

### Cohorts for the Study of Viral Control

The existence of clinical cohorts/studies prospectively collecting data and samples from individuals with HIV makes it more straightforward to study post-infection outcomes. Various measures of HIV-1 control and disease progression have been used as phenotypes in host genetic studies (Box 1). Plasma viral load and CD4+ T cell count are routinely collected during clinical follow-up and are thus widely available for large numbers of patients. These markers have been shown to be independent predictors of progression to severe immunodeficiency [12]. Later measures of progression like AIDS-defining events, specific opportunistic infections, or AIDS-related death are more likely to result from complex interactions between multiple genetic and environmental factors: the power to detect true genetic effects is thus reduced. In addition, later outcomes can only be assessed in historical cohorts, thanks to the efficacy of current antiretroviral treatments. The study of the earliest stages of infection represents an especially challenging task, since identification and recruitment of acutely infected patients is hampered by significant scientific and sociologic limitations. Finally, it is important to note that, so far, the vast majority of HIV-1 host genetic studies focused on patients of Western European ancestry, in striking contrast with the global distribution of HIV-1 burden. The creation and analysis of cohorts in other ethnic groups is clearly a priority for ethical reasons, but also because population diversity increases the likelihood of genetic discovery.

**Box 1. Phenotypes That Have Been Used in Genetic Studies of HIV-1 Infection**

HIV resistance/acquisition:

- Mucosal exposure
- Intravenous exposure
- Mother-to-child transmission

HIV viral load:

- Set point viral load
- Intracellular HIV-1 DNA level
- Viral control (elite control/viremic control)

HIV disease progression:

- Slope of CD4+ T cell decrease
- Time to CD4+ T cell decrease below a certain threshold
- Time to AIDS 1987/AIDS 1993
- Time to death
- Long-term non-progression
- Rapid disease progression

### Cohorts for the Study of Therapeutic Outcomes

Human genomic approaches can also be used to study therapeutic intervention outcomes. Although antiretroviral therapy is highly effective, critical questions remain about how patients respond to treatment: genetic variation among individuals and populations may cause considerable variability in drug pharmacokinetics and pharmacodynamics. In particular, since anti-HIV medicines are used for life, even modest differences in susceptibilities to adverse events can become important. Identification of risk profiles could allow the tailoring of the drugs to minimize the long-term toxicities associated with treatment, like metabolic disturbances and cardiovascular diseases. Consequently, an important effort should be made to obtain informed consent for genetic testing from participants in randomized clinical trials, which represent an ideal setting for pharmacogenetic discovery, as recently demonstrated, for example, in hepatitis C research [13], [14]. Genetic variation will also be responsible for differences in vaccine immunogenicity and tolerability, and as such needs to be considered in the design and evaluation of current and future HIV-1 vaccine trials.

## Common Variation and the Control of HIV-1

Most recent HIV-1 host genetic studies have interrogated human genetic variants for their association with viral load (either as a continuous trait, or as a categorical variable in studies of controllers) and CD4+ T cell decrease. Only a few confirmed genetic associations are understood in terms of the responsible causal sites, and even if it is the case, fundamental questions remain about the exact mechanisms involved in viral control. Most prominent is the example of human leukocyte antigen (HLA) class I variation, and notably of HLA-B*5701, whose protective effect has been shown to be the largest contributor to inter-individual variability for both viral set point and CD4+ T cell decline in genome-wide association studies performed in populations of recent European ancestry [3]–[7]. Interestingly, a very similar result has also been observed in a genome-wide study performed in African-Americans, where HLA-B*5703 was the most important determinant of viral control [9]. *HLA-A*, *-B* and *-C* are extremely polymorphic and encode protein products that are fundamental in the immune recognition process: expressed at the cell surface, they present antigenic epitopes including processed viral peptides to CD8+ T lymphocytes, thereby initiating a cytotoxic T cell (CTL) response. Other HLA-B types have been shown to associate with differences in HIV-1 outcomes [15]: HIV-1 control is better in the presence of HLA-B*27, B*51, and B*5801, but poorer in the presence of HLA-B*5802 and of alleles from the HLA-B35Px group [16]–[19]. Homozygosity for class I alleles also leads to faster progression and higher viremia, presumably because it reduces the diversity of the epitope recognition machinery, thereby impairing antiretroviral CTL response [16].

Beyond the key role they play in the induction of CTL responses, HLA class I molecules are also ligands for the killer cell immunoglobulin-like receptors (KIRs), expressed at the surface of natural killer (NK) cells. KIRs regulate NK cell activation status through inhibitory or activating signaling and can thereby have a direct modulating effect on the innate immune response to HIV-1 infection. Certain combinations of KIR genes and HLA class I alleles have epistatic influences on the outcome of HIV-1 infection [20]: *KIR3DL1* and *KIR3DS1* have been associated with better control of HIV-1 when they are found in patients that have HLA-B alleles with a Bw4 specificity. Recently, we showed that copy number variation of the *KIR3DL1/KIR3DS1* locus results in differences in HIV-1 control in the presence of HLA-Bw4 (K. Pelak, A. C. Need, J. Fellay, K. V. Shianna, S. Feng et al., unpublished data).

Other genetic associations, albeit statistically unequivocal, are still poorly understood. A polymorphism located in the upstream region of *HLA-C* (HLA-C -35) associates with both HIV-1 control and expression levels of the gene [3], [21], suggesting that the number of HLA-C molecules expressed at the cell surface might play a role in the efficacy of the immune response. Genome-wide studies also detected additional independent associations in the major histocompatibility complex (MHC) [3]–[5], [7], but the long-range linkage disequilibrium structure of the region makes it virtually impossible to pinpoint the real causal sites using genetic data alone.

Outside of the MHC, nearly all genetics findings reported to date resulted from candidate gene studies. As a consequence, variants were identified in genes implicated in HIV-1 life cycle or in immune-related genes. The problem, however, is that most results are equivocal or controversial, due to technical or methodological limitations. In particular, the quasi-systematic absence of correction for population stratification before the genome-wide era has been responsible for a high number of false positive results [7], [22]. In fact, other than HLA and KIR variation, only polymorphisms located in the chemokine receptor cluster on chromosome 3 have been repeatedly associated with HIV-1 control: specifically, heterozygosity for a 32–base pair deletion in *CCR5* (CCR5Δ32), variants of the *CCR5* promoter region, and a non-synonymous coding change in *CCR2* (V64I) have been consistently shown to associate with differences in viral load and/or disease progression [7], [23]–[28].

## Common Variation and Acquisition

Variation in *CCR5* remains the only human genetic determinant that has been proven to significantly impact HIV-1 acquisition: both the CCR5Δ32 variant (present in homozygous form in about 1% of Europeans) and the m303T>A point mutation (much rarer) result in a defective CCR5 protein product that is not expressed at the cell surface [23], [29]–[31]. When present in homozygous or combined heterozygous form, they confer complete resistance to infection by HIV-1 viruses that use CCR5 as co-receptor. Of note, those individuals remain susceptible to infection by CXCR4-using viruses (including dual-tropic viruses) that associate with more rapid HIV disease progression [32].

Other gene variants were reported to protect against acquisition or to increase susceptibility to infection, but they are at best supported by weak evidence from candidate gene studies. In fact, none could yet be replicated using contemporary standards widely accepted in human genetics, notably a correction for population stratification: for example, we and others reported a lack of association between HIV-1 susceptibility and the number of copies of *CCL3L1* [33]–[35], or the allelic distribution of a *DARC* promoter variant [36]–[40].

Given the importance of the natural model of resistance to HIV-1 infection, it comes as a surprise that no genome-wide study has been published that looks at correlates of protection. It is clearly a priority for the HIV genetic field to carry out such studies.

## The Role of Genetic Variation: The Complete Picture

**Figure 1:**
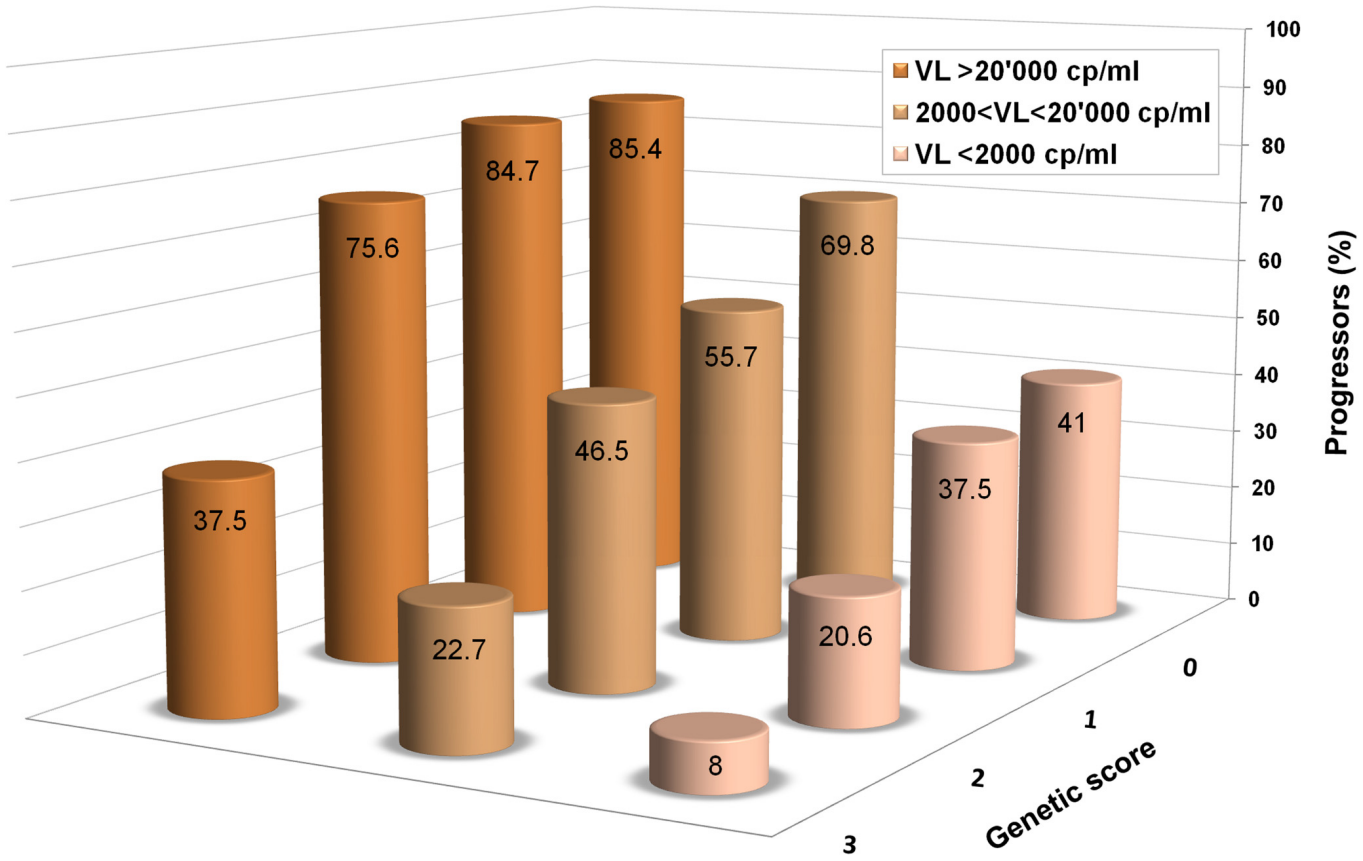
An additive genetic score helps predict HIV-1 disease progression. Data are from Fellay et al. [7]: 1,071 individuals of Caucasian ancestry with HIV-1 are included in the analysis. The columns show the proportions of individuals that reached a progression outcome (CD4+ T cells <350/ul or initiation of combined antiretroviral treatment with CD4+ T cells <500/ul) during the first 5 years after estimated date of seroconversion in categories defined by HIV-1 viral load and by a simple additive genetic score, in which one unit is counted for each “protective” allele. The minimum score is 0 for individuals that are homozygous for the major allele at rs2395029 (a proxy for HLA-B*5701), rs9264942 (*HLA-C* -35 variant), rs9261174 (*ZNRD1*), and CCR5-Δ32. The maximal observed score is 3 since no individual was heterozygous or homozygous for the minor allele at all four sites. Individuals were grouped in three categories to clearly show that the genetic score refines the prediction of progression, beyond the information provided by viral load only, throughout the range of set point values.

It seems reasonable to conclude that most of the common variants important in the control of HIV-1 have now been identified, at least in individuals of European ancestry. Despite this, it appears that most of the inter-individual differences in control remain to be explained. The confirmed host genetic determinants of HIV-1 control are only able to explain about 20% of the observed variation in viral load or disease progression [7]. It is noteworthy that such limited genetic knowledge can already be used to refine the prediction of disease progression, beyond the information provided by viral load only, as shown in Figure 1.

So, what is responsible for the large variability in HIV-1 control that still remains unexplained? Clearly, part of it is attributable to the virus itself, as demonstrated by sudden changes in disease course and/or viral set point upon super-infection in chronically infected patients [41] and by sizeable differences in viral load set point that can be observed between the donor and the recipient in HIV-1 transmission pairs [42]. Environmental influences also play a role: for example, pro-inflammatory diseases are often associated with a significant increase in HIV-1 viral load, while co-infections with viruses like GB virus C, HTLV-1, and HIV-2 have been reported to have an inhibitory effect on HIV-1 replication [43].

However, as it is the case for many other human complex traits, it is not unreasonable to assume that rarer genetic variants are responsible for a sizeable fraction of the unexplained inter-individual differences [44]. For example, it is clear that a fraction of the population is highly resistant to infection by HIV-1. While homozygosity for CCR5Δ32 is responsible for some of these cases [23], [29], [30], it appears to explain only a minority of such observations. Several studies have shown a higher frequency of CCR5Δ32/Δ32 in HIV-uninfected hemophiliacs than in the general population (up to 25% compared to 1%, respectively), with the highest frequencies in those with severe hemophilia [45]–[47]. Homozygosity for CCR5Δ32 is also significantly enriched in highly exposed, yet seronegative homosexual men [23], [24]. While those numbers clearly illustrate the high degree of exposure in these populations, it also suggests that other protective mechanisms are responsible for most individual cases of resistance. So far, genome-wide studies in these groups also fail to reveal any strong common variants conferring further protection (D. Goldstein, unpublished data). The identification of the other variants responsible for protection therefore will require a deeper interrogation of the human genome than is possible using genome-wide association studies.

**Figure 2:**
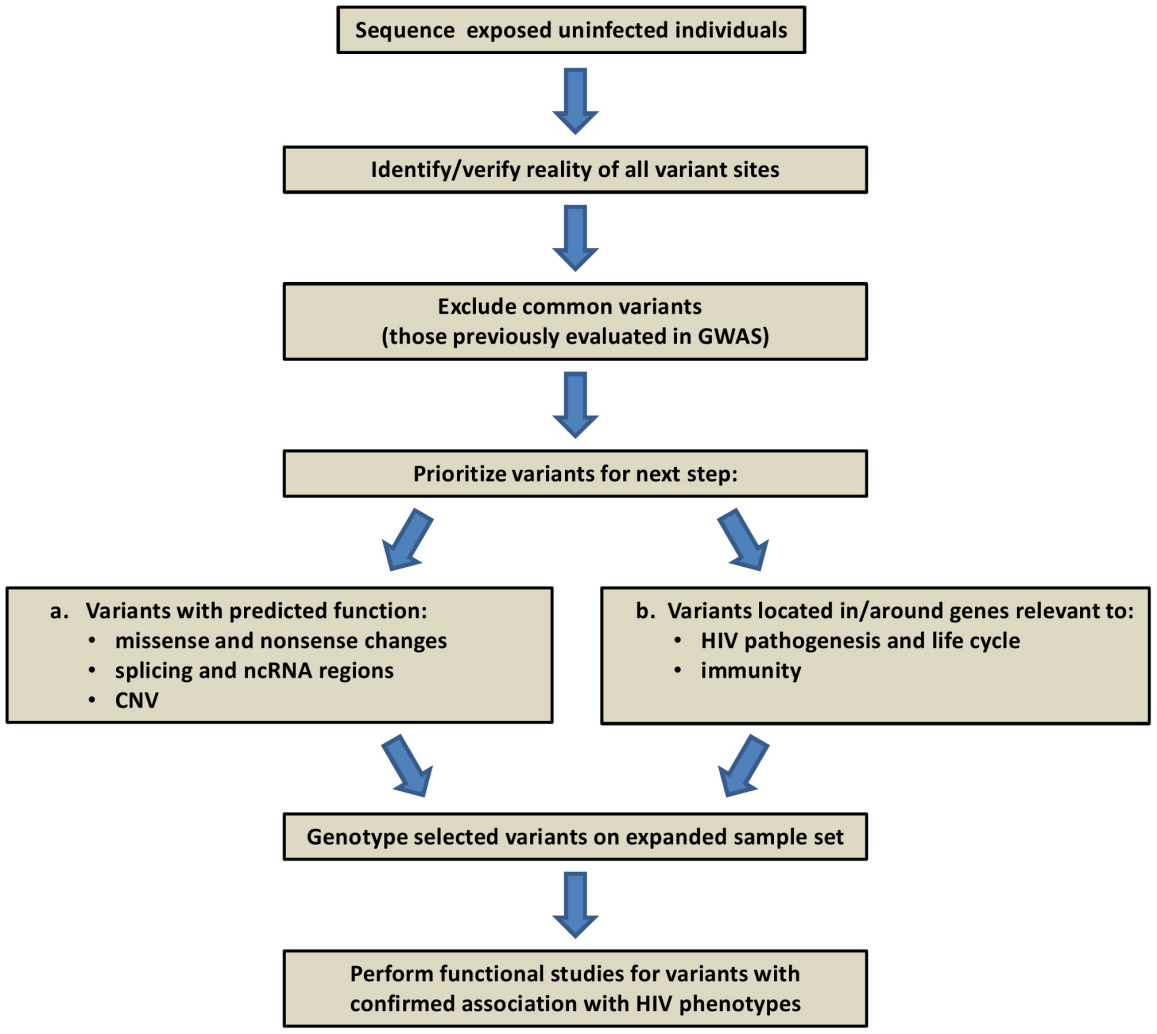
Project framework: human genome resequencing of hemophilia A individuals exposed to HIV-contaminated factor VIII in 1979–1984, yet uninfected.

While still expensive and difficult to implement due to computational and bioinformatic challenges, it is feasible to carry out systematic discovery genetics using whole exome or whole genome sequencing [48]. Several recent reports demonstrated that the cause of Mendelian diseases can be identified using such resequencing strategies [49]–[51]. In the HIV field, one project already underway involves sequencing the complete genomes of 50 hemophilia patients (Figure 2). Discovery of variants in this framework will depend principally on three factors: 1) the initial population frequency, 2) the degree of enrichment in frequency in the exposed uninfected individuals, and 3) the defining characteristics of a rare causal variant, e.g., predicted functional consequence, clustering in a gene/pathway, conservation. Whatever the ease of recognition, it seems reasonable to expect that causal variants can be identified using a combination of sequencing in a discovery cohort and confirmation by genotyping in a much larger validation cohort.

Other phenotypes related to HIV control that could be studied using a similar design include extremely rapid versus extremely slow progression, as well as resistance to infection through mucosal exposure. Other more complicated phenotypes may also be interesting targets for study, such as the occurrence of persistently high viral loads without progression to AIDS [52], extreme levels of microbial translocation during acute infection [53], or unusual immune activation patterns (Box 2).

**Box 2. Examples of Target Phenotypes for Human Genome Resequencing Studies**

- Resistance to HIV-1 infection in highly exposed uninfected individuals
- Very rapid disease progression
- Elite viral control
- Poor viral control in HLA-B*57 individuals
- Persistently high viral loads without apparent disease progression
- Degree of intestinal microbial translocation during acute infection
- Unusual immune activation patterns

## Systems Approach

**Figure 3:**
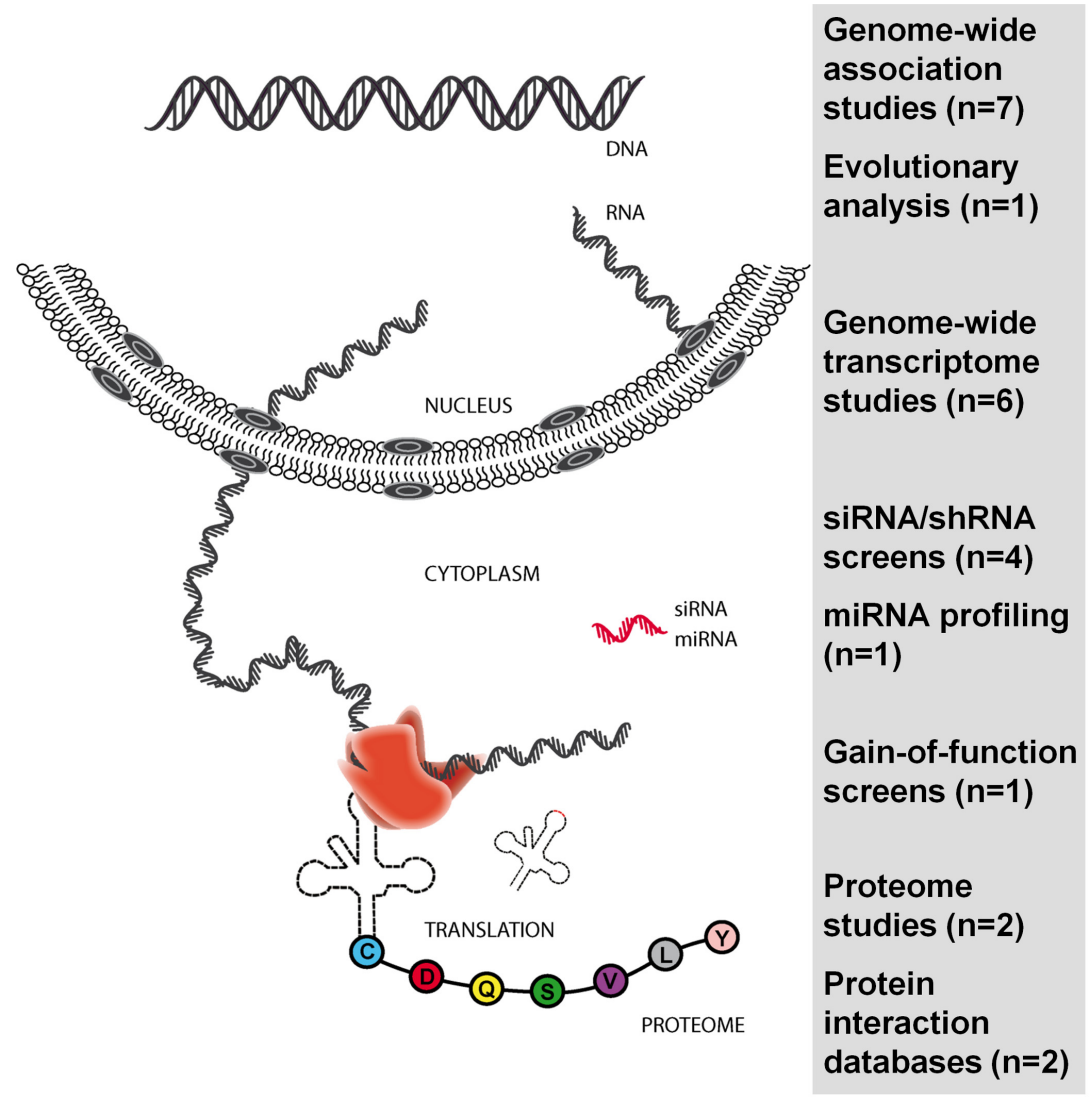
Genome-wide and large-scale studies published since 2007 in the HIV field. The number of studies is in parentheses. Diverse sets of results and data are compiled in an encyclopedia of overlaps between studies (http://www.hostpathogen.org/). This approach serves to identify networks used by HIV-1 to support its replication. Figure updated from reference [55] (http://F1000.com/Reports/Biology/content/1/71).

Beyond the information generated by genome studies, the field can now press ahead with novel approaches that use a range of technologies. Prominent among these are the analyses of the transcriptome and proteome, and small interfering RNA (siRNA) screens (Figure 3). These genome-wide studies generate large data sets that can be analyzed in isolation, and, increasingly, in an integrated manner [54], [55]. Below we summarize key studies in the HIV field using these techniques, and the first efforts at feeding information across studies. The last section will address the prospects for a systems biology approach in the study of HIV-1 biology and pathogenesis.

### Transcriptome Analyses

New microarray technologies have recently allowed the genome-wide analysis (>≈20,000 transcripts) of infected cells in vitro, and in vivo in individuals with HIV [56]–[59]. Dynamic analyses have been also completed in animal models. The cell types investigated varied from the collective study of peripheral blood mononuclear cells, to cell type–specific studies [60]. The overarching messages from these studies are (i) the massive modulation of the antiviral defense systems (the interferon response, including the antiretroviral intrinsic cellular defense apparatus), (ii) the prominent modulation of genes involved in the cell cycle and degradation/proteasome pathways, and (iii) the absence of a characteristic expression pattern of effective control of viral replication (e.g., in elite controllers). Evidence of a persistent deregulated interferon response upon infection is of particular interest in light of comparative studies of pathogenic and non-pathogenic animal models [61], [62]. Upon primary simian immunodeficiency virus infection of sooty mangabeys and of African green monkeys, these natural hosts display a strong interferon response at seroconversion followed by distinctive down-regulation despite persistence of ongoing active viral replication [63], [64]. The precision of transcriptome analyses will be greatly improved through the added resolution of RNA-Seq [65] and the capacity to look at the transcriptome in single cells [66].

### Proteome Analyses

Large-scale studies are limited by the number of proteins that can be assessed in a quantitative fashion. Analyses of 2,000 to 3,200 proteins identified 15%–21% to be differentially expressed upon infection [67], [68], including changes in the abundance of proteins with known interactions with HIV-1 viral proteins. The NCBI HIV-1 Human Protein Interaction Database (http://www.ncbi.nlm.nih.gov/RefSeq/HIVInteractions/) summarizes over 3,000 interactions with almost 1,500 human genes [69]. Other datasets of interest include the human–pathogen protein–protein interactions (PPIs)/pathogen interaction gateway (PIG) [70] that reports that pathogens tend to interact with hubs (proteins with many interacting partners) and bottlenecks (proteins that are central to many paths in the network) in the human PPI network [71]. No integrated approaches have been used so far to analyze these data in the context of other genome-wide studies.

### siRNA and Gain-of-Function Screens

**Figure 4:**
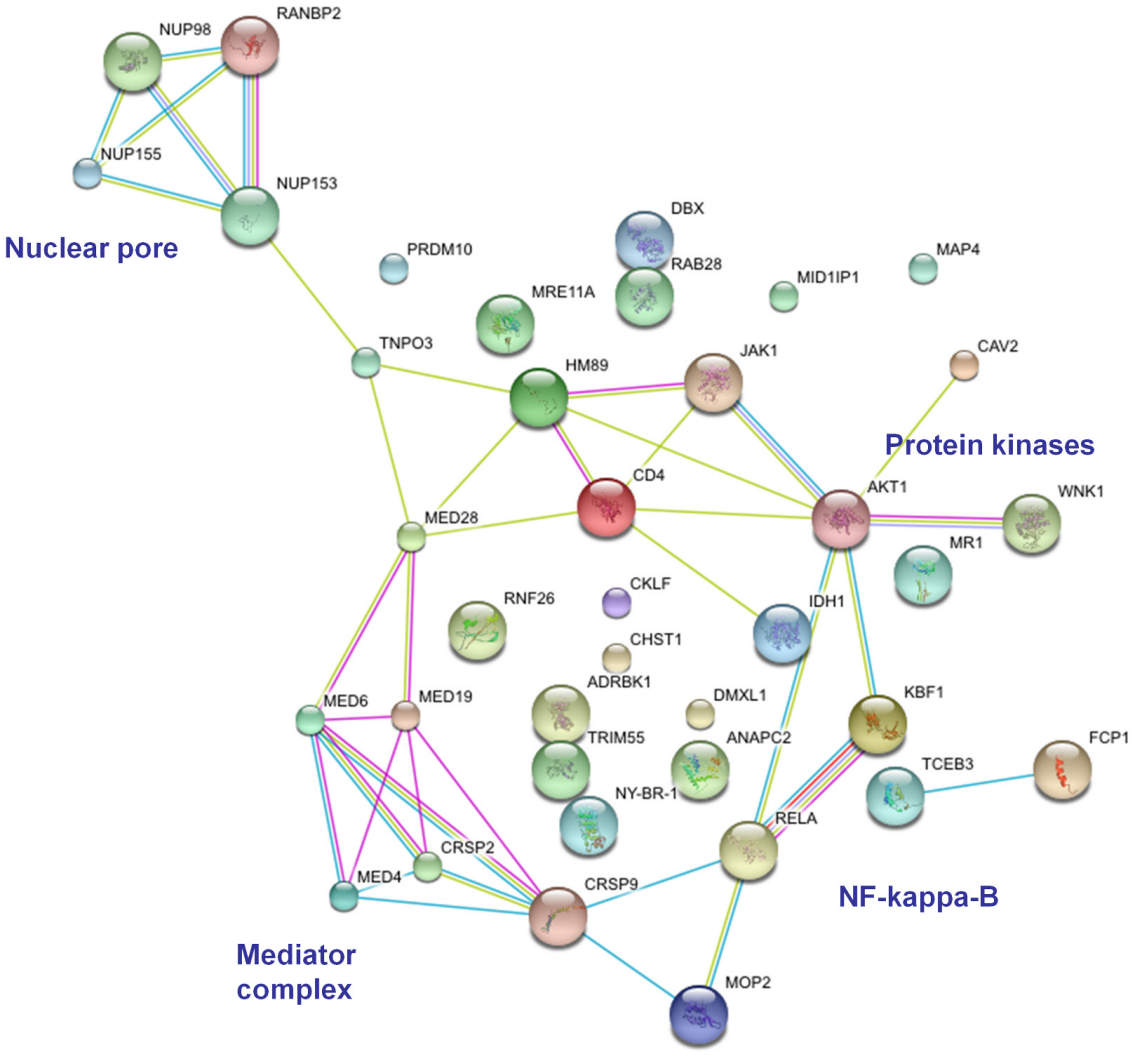
Predicted interaction networks of genes identified as HIV dependency factors in siRNA/shRNA screens [72]–[75]. Links have been predicted using STRING (http://string.embl.de/). Predicted interactions are depicted according to the type of available evidence. The interactions (see color labels) include direct (physical) and indirect (functional) associations; they are derived from four sources: genomic context, high-throughput experiments, conserved co-expression, and previous knowledge from literature. The nature of the supporting evidence is indicated by the color lines: yellow, text mining; purple, experimental; red, gene fusion; light blue, protein–protein interactions; blue, genomic co-occurrence evidence.

Three siRNA transfection [72]–[74] and one short hairpin RNA (shRNA) [75] transduction studies have targeted the coding RNA for >20,000 human proteins. Approximately 1,000 proteins have been identified as potentially necessary for an optimal viral replication. However, there was minimal overlap across studies—possibly because of differences in cell types and in study design. None of the studies captured or were designed to identify genes that would restrict viral replication—i.e., their silencing would result in greater viral production. Overall, 34 genes were identified in two or more of the transfection screens. However, among those genes that were shared by one or more studies, a pattern emerged that involves the nuclear pore machinery, the mediator complex, a number of key kinases, and components of the NF-κB complex (Figure 4). One gain-of-function screen used a cDNA library representing 15,000 unique genes in an infectious HIV-1 system [76]. This led to the proposal of novel proviral host factors.

### Evolutionary Data

Genes involved in immunity and inflammation among those exhibiting the strongest signatures of positive selection both across species and within humans [77]–[84]. Increasingly, evolutionary and comparative sequence analyses across species or within human populations can be used to identify genes that have played a major role in host survival and are therefore likely to influence modern susceptibility to, or pathogenesis of, infectious diseases [78], [85]–[87]. Given the relevance of endogenous and exogenous retroviruses in primate evolution, the identification of genomic signatures can provide an additional layer of data for analysis of contemporary susceptibility to HIV-1. A number of targeted analyses of genes involved in cellular defense against retroviruses have been reported [88]–[92]. A systematic study of long-acting selective pressures on primate genomes (analysis of 140 genes proven or possibly involved in HIV-1 biology and pathogenesis) reached the following conclusions: (i) there are three general groups of genes presenting different evolutionary histories of their coding regions in primates, (ii) analyses allow a non a priori identification of candidate residues that affect host–pathogen interactions, and (iii) a subset of genes may remain under positive selective pressure in modern human populations [92].

### Data Integration

Progressively, researchers aim at integrating different layers of data. Rotger et al. [59] examined the correspondence of results from genome-wide transcriptome analysis of differentially expressed mRNA in CD4 T cells from infected individuals with results from analysis of *cis*-acting genetic variants modulating gene expression in the same samples. In this work, 265 genes were differentially expressed in CD4 T cells across the range of viral set point, and 160 genes were shown to have *cis*-acting genetic variants associated with expression. However, the overlap between the two lists was minimal: only one gene was common to both lists: *OAS1*, an interferon-stimulated gene. However, SNPs in this gene are not associated with notable differences in viral set point or disease progression.

Bushman and colleagues [54] applied meta-analytical procedures to assess a wider range of genome-wide studies and public interaction databases. A higher level of signal would be obtained if the data were evaluated in the frame of specific networks and cellular systems. The approach led to the identification of at least 11 densely connected clusters. These clusters, which are enriched for proteins identified in multiple separate screens, specify cellular subsystems associated with HIV replication: the proteasome, subunits of RNA polymerase II and associated factors, the mediator complex, the Tat activation machinery, RNA binding and splicing proteins, and the BiP/GRP78/HSPA5 and CCT chaperones. The study went one additional step to organize data in an “encyclopedia” of host factors assisting HIV replication (http://www.hostpathogen.org/).

There is growing interest in applying non-reductionist approaches such as systems biology to the study of infectious diseases. The general premise of systems biology includes the high-throughput quantitative approach to a biological system that can be subjected to iterative cycles of perturbation, and the modeling of the collected data. HIV infection, which results in a perturbed environment that can be exogenously manipulated through treatment, or modulated by genetic determinants, should now be approached under this research paradigm.

## Conclusion

The aim of HIV host genetic research is to comprehensively describe human genetic influences on HIV/AIDS. Some genetic factors have now been convincingly associated with viral control or resistance to infection, yet much effort is still needed to get the full picture. The field is now moving simultaneously towards greater depth in genome analysis and towards more breath and integration through systems biology. This ongoing transition brings renewed hopes that genetic analysis of the human host will contribute substantially to understanding HIV-1 pathogenesis and developing new strategies to stamp out the AIDS pandemic.
